# Case report: A rare cause of chest pain: Kommerell's diverticulum

**DOI:** 10.1016/j.ijscr.2021.106323

**Published:** 2021-08-19

**Authors:** Syed Muhammad Azeem, Umer Arif, Raziullah Baig, Mohammad Iqbal Khan

**Affiliations:** a Shifa International Hospital Islamabad, Pakistan; b Shifa Tameer-e-Millat University, Pakistan

**Keywords:** Aberrant right subclavian artery, Kommerell's diverticulum, Subclavian artery aneurysm

## Abstract

**Introduction and importance:**

Kommerell diverticulum is a very rare congenital defect of the aortic arch associated with the aberrant subclavian artery. It can present with signs of dysphagia, chest pain, or distal embolization in the upper limb.

**Case presentation:**

We present a case of Kommerell diverticulum with associated large subclavian artery aneurysm in a male patient with chest pain of unknown origin and hypertension. There was an incidental finding of the wide mediastinum on chest X-ray and the patient had a difference in systolic blood pressure in both arms. A right thoracotomy incision was used to successfully excise the aneurysm and reconstruct the subclavian artery. Patient recovery was uneventful.

**Clinical discussion:**

Endovascular approaches are also an alternative to conventional open surgeries in the treatment of Kommerell diverticulum.

**Conclusion:**

Kommerell diverticulum with subclavian artery aneurysm should be considered in the differential diagnosis of non-cardiac chest pain. A simple investigation such as a chest X-ray can make a difference in these patients. Coarctation related to the right ASA might not always be a true coarctation. Endovascular treatment is an alternative to open repair in selected cases, but it needs further investigation in large randomized control trials.

## Highlights

• Kommerell's diverticulum (KD) is an extremely rare developmental abnormality with prevalence of right aberrant subclavian artery originating from the left aortic arch has been reported to be 0.7%–2.0%.
• The catastrophic events of aneurysm rupture and aortic dissection has been reported to be 53% and 19% respectively.
• aggressive surgical treatment is suggested for KD larger than 3cm at the origin and symptomatic aneurysms larger than 5 cm.
• All symptomatic patients with compressive symptoms should undergo open repair rather than endovascular repair.
• Coarctation of aorta is associated in 1 % of cases of right aberrant subclavian artery while only 150 cases of pseudocoarctation has been reported in literature.

## 1 Introduction

Kommerell's diverticulum (KD) is an extremely rare developmental abnormality of the aortic arch related to an aberrant subclavian artery. KD is also known as “lusoria diverticulum,” referring to the aneurysmal dilatation of the descending aorta at the origin of an aberrant subclavian artery (ASA) [1]. The right subclavian artery (SA) usually originates from the brachiocephalic artery, but in 0.4–1.8% of the general population, it may arise directly from the aortic arch distal to the left subclavian artery [2]. Right ASA with aneurysm (KD) is more likely to have pressure symptoms (dysphagia, dyspnea, coughing, chest pain, rupture, right upper extremity ischemia) [3]. Most of these aortic abnormalities are discovered incidentally on CT scans performed for other reasons. Due to its rare presentation, the natural course of the disease is not well understood. The catastrophic events of aneurysm rupture and aortic dissection has been reported to be 53% and 19% respectively. Hence, surgical management is advised in most cases [3].

## 2 Case report

**Fig. 1:**
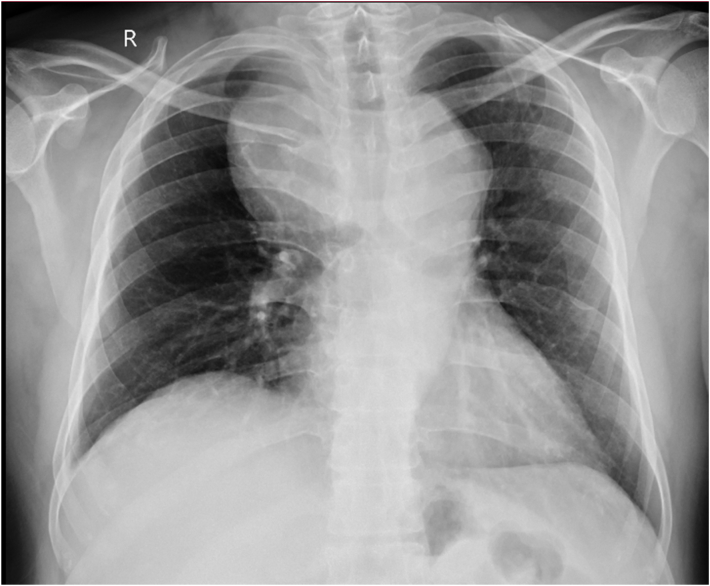
Chest X-ray of the patient showing a wide mediastinum.

**Fig. 2:**
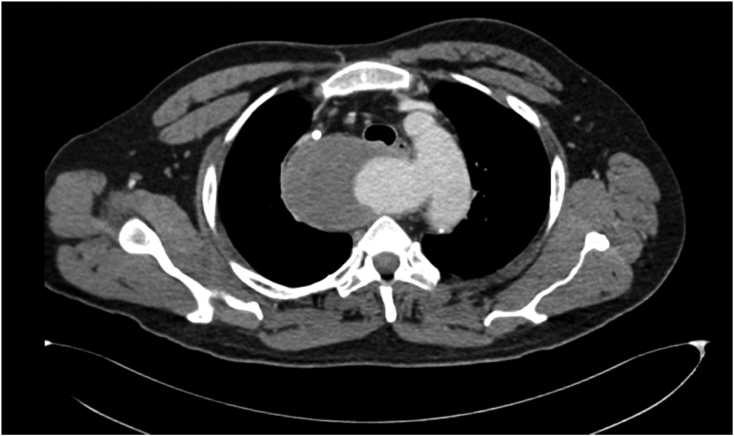
CT scan of the patient showing Kommerell's diverticulum.

**Fig. 3:**
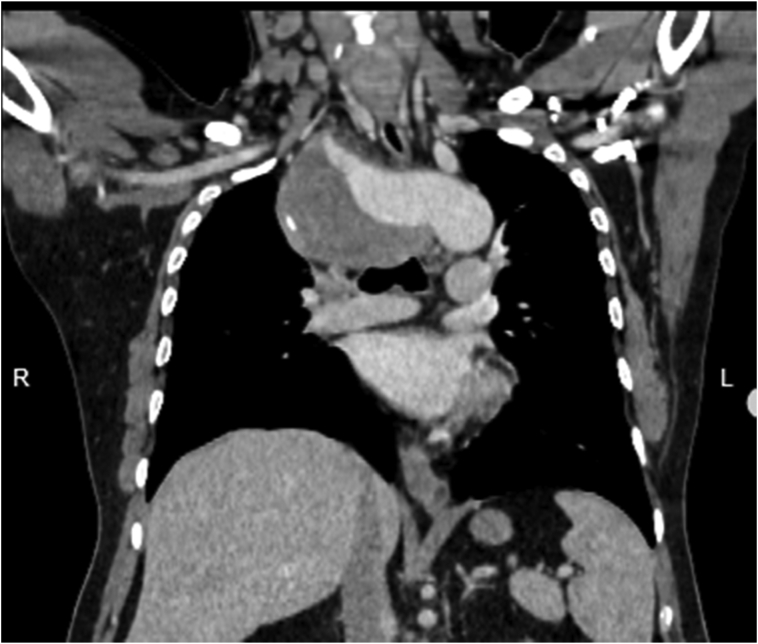
Axial view showing Aberrant right subclavian artery aneurysm.

A 47 years old gentleman presented to the surgical clinic with complaints of undiagnosed chest pain for 3 months. The pain was dull in character and insidious in onset. It was localized to the central chest and radiating to the back. He had no complaints of dysphagia or indigestion. He had a significant medical history of uncontrolled hypertension and end-stage renal disease. He was on loprin 75 mg, atenolol 100 mg, and hemodialysis. His past surgical history was consistent with bilateral DJ stenting and PCNL (percutaneous nephrolithotomy) for renal stones. He had no significant family history of hypertension, diabetes, or cardiac conditions. His psychosocial history signified elements of stress and depression related to his recent loss of a child. The patient's baseline investigations showed anemia with raised creatinine and CRP. ECHO was normal with an ejection fraction of 60%. He also had angiography performed for uncertain chest pain with no significant findings Physical examination revealed a systolic blood pressure difference of 10 mmHg in both arms (Table 1). The right radial pulse was not palpable. Chest X-ray (Fig. 1) showed a wide mediastinum. CT angiography (Fig. 2, Fig. 3) of the chest was advised which showed the aberrant origin of the right subclavian artery with a 36.4 × 68.4 mm aneurysm. The aneurysm was partly thrombosed and compressing the right internal jugular vein. The ASA was originating from a 28 mm Kommerell's diverticulum and there was a coarctation present at the junction of the arch and descending thoracic aorta measures approximately 21 mm. Bilateral carotids left subclavian and rest of the visualized thoracic aorta was unremarkable and patent. The right subclavian artery was patent originating directly from the arch of the aorta. The right renal artery was attenuated with an atrophic right kidney. The vascular surgeon and anesthesiologist counseled the patient in detail about the risks and adverse events during and after the procedure. His preoperative investigations were performed, review with the anesthesia team and cardiac surgery team was conducted. The endovascular approach was unsuitable in this patient because of the patient young age, no proximal landing zone, and a large symptomatic right ASA aneurysm with a high risk of rupture and distal right upper limb ischemic embolism. A vascular surgeon performed the surgery. The right thoracotomy approach was used to dissect aneurysm from surrounding tissue, KD was clamped and oversewed, and the right subclavian artery was reconstructed using an 8 mm Dacron graft. The coarctation was found to be pseudocoarctation, due to the right ASA aneurysm pressing on the arch of the aorta. Postoperative recovery was uneventful. The patient was kept in a surgical ICU for 2 days. He remained stable in the ward with no signs of cardiovascular or respiratory compromise. He was mobilized out of bed on the second postoperative day and discharged on the fifth postoperative day. This case report has been reported in line with the SCARE 2020 criteria [14].

**Table 1:** Pulse and blood pressure measurements in four limbs.

	Right arm	Right leg	Left arm	Left leg
Blood pressure/mmHg	90/70	150 systolic	160/80	150 systolic
Ankle brachial pressure index	1.6		0.9	
Pulse	Not present	Present	Present	Present

## 3 Discussion

A German Radiologist ‘Kommerell’ first described this pathology in 1963 while studying a barium swallow and discovering a pulsatile mass posterior to the esophagus causing its compression [4]. KD is a congenital anomaly of the aortic arch that arises due to failed involution at the fourth primitive dorsal arch. KD is present in approximately 20%–60% cases of ASAs. The complications associated with KD are diverticular rupture (4%), aortic dissection (11%), aortic aneurysm rupture, or ASA aneurysm rupture [5]. Conservative management is appropriate for asymptomatic patients with aberrant right subclavian artery without a Kommerell diverticulum. The presence of symptoms or aneurysmal degeneration should prompt treatment [3]. There are no general guidelines for the management of KD associated with a large right ASA aneurysm. One study has advocated aggressive surgical treatment for KD larger than 3 cm at the origin while another study has suggested surgical treatment for symptomatic aneurysms larger than 5 cm [4].

The surgical options of repair and construction of the right ASA include left thoracotomy with side clamping and repair oversewing of the origin of the right SA plus carotid subclavian bypass/interposition, or median sternotomy with the reconstruction of the right SA using a tube bypass from the ascending aorta, or repair of the aorta with interposition graft [5], [6], [7].

Coarctation of the aorta can be present in 1% of the patients with right ASA [8] while approximately 150 cases of pseudocoarctation have been reported in literature [9].

Surgical procedures can be more demanding in patients with multiple comorbidities with large diverticula or a greatly dilated aorta. An endovascular repair is an alternative approach to surgical repair with fewer complications but it is unsuitable for patients having compressive symptoms. In a case series by Bloom et al., 31 patients underwent open repair (thoracotomy) while 12 patients had an endovascular repair. All symptomatic patients with compressive symptoms underwent open repair while asymptomatic or those presenting in emergency underwent endovascular repair. Mid-term mortality was higher in the endovascular group with two patients having a stroke. There were no statistically significant differences in other postoperative complications or hospital length of stay between the two groups [10]. KD has a female predominance with a growth rate of 1.45 ± 0.39 mm/year [11]. Due to its slow growth rate asymptomatic patients can be followed with serial CT scans [12]. According to a systemic review endovascular repair was used to treat both symptomatic and asymptomatic cases with safe and favorable results. Thrombosis and shrinkage of the aneurysm were noted in 71% of patients on follow-up CT scans [13].

KD is a rare condition associated with ASA degeneration. Endovascular treatment is an alternative to open repair in selected cases, but it needs further investigation in large randomized control trials. Coarctation related to the right ASA might not always be a true coarctation. The radiological images of such cases should always be discussed in detail with the radiologists, keeping in mind the possibility of pseudocoarctation.

## Ethical approval

Granted by Institutional review board and ethics committee (IRB/EC) of Shifa International Hospital.

## Sources of funding

Self-finance

## CRediT authorship contribution statement

Dr. Syed Muhammad Azeem: Conception, design, and drafting the article. First assistant to a vascular surgeon.

Umer Arif: revision of the article, second assistant

Razi Ullah Baig: revision of the article

Prof M. Iqbal Khan: revised and approved manuscript.

## Guarantor

Prof M. Iqbal Khan, Professor of Vascular Surgery. Shifa Tameer-e-Millat University.

## Research registration

Not required.

## Provenance and peer review

Not commissioned, externally peer-reviewed.

## Consent

Written informed consent was obtained from the patient for publication of this case report and accompanying images.

## Declaration of competing interest

None.
